# Influence of Design Parameters of Membrane-Type Flow Controller on Bearing Characteristics of Hydrostatic Guideways

**DOI:** 10.3390/mi16080891

**Published:** 2025-07-30

**Authors:** Yi Chen, Xiaoyu Xu, Ziqi Lin, Maoyuan Li, Guo Bi, Zhenzhong Wang

**Affiliations:** 1 School of Aerospace Engineering, Xiamen University, Xiamen 361005, China; 2 Sichuan Institute of Xiamen University, Chengdu 610213, China

**Keywords:** flow controller, hydrostatic guideways, fluid–solid coupling simulation, bearing characteristics

## Abstract

The hydrostatic guideway has been widely used in ultra-precision machine tools. The flow stability of the hydrostatic guideway has a significant impact on its bearing characteristics, and the flow controller is critical to safeguard the flow stability of the hydrostatic guideway. Currently, most engineering applications use fixed, fluid-resistance flow controllers, which have a simple structure, low cost, and high reliability. However, when facing complex working conditions, the fixed, fluid-resistance flow controller cannot maintain the flow stability of the hydrostatic guide. In this study, a membrane-type flow controller with variable fluid resistance is designed, and a theoretical model of the flow controller’s bearing characteristics is established, which is verified by fluid–solid coupling simulation and flow rate experiments. Analyzing the influence of the design parameters of the membrane-type flow controller on the performance according to the theoretical model, the design guidelines of the membrane-type flow controller are established, the key structure of the flow controller is clarified, and the design range of the key structure dimensions is given. The results show that the gasket thickness of the membrane-type flow controller has the greatest impact on the performance of the hydrostatic guideways, which should be ensured to have a machining error of less than 0.005 mm. This study is a guide for the design and manufacture of flow controllers, as well as for engineering applications.

## 1. Introduction

The hydrostatic guideway has been widely used in ultra-precision machine tools due to their high running accuracy [1], high stability [2], excellent damping characteristics [3], low friction [4], and long life [5]. The working principle of the hydrostatic guide is that the hydraulic pump pressurizes the hydraulic oil and then conveys it to the oil pocket of the hydrostatic slider through the pipeline. After the high-pressure oil enters into the oil pocket, it overflows outward from the tiny gap between the guide and the slider (oil sealing surface), forming a layer of uniform hydrostatic oil film [6]. In hydrostatic guideways, the oil film maintains a sufficient stiffness to ensure load-bearing capacity and precision. The thickness and stiffness of the hydrostatic oil film are jointly controlled by the oil pump pressure, hydraulic oil viscosity, load, and flow controller. Hydrostatic guideways are subjected to varying loads during operation, especially from the continuous reduction in the workpiece weight during material removal, fluctuations in the grinding force caused by wheel cut-in, cut-out, and wear, and the uneven hardness of the workpiece material. The variation in the loading results in a change in the hydrostatic oil film thickness, which, in turn, leads to a change in the relative position of the workpiece and the tool. Therefore, the load-bearing characteristic is a critical performance index for hydrostatic guideways, and scholars have conducted a series of studies to enhance the bearing characteristics of hydrostatic guideways, for instance, by changing the shape of the oil pocket [7,8], optimizing the distribution of the oil pocket [9,10], reducing the oil film thickness [11], texturing the surface of the guideway [8,12], and applying a microgroove structure on the working surface of hydrostatic bearings [13]. The flow rate stability of the hydrostatic guideway has a significant impact on its load-bearing characteristics, and the flow controller is the key to guaranteeing the flow rate stability of the hydrostatic guideway. Currently, most engineering applications use fixed, fluid-resistance flow controllers, such as small-hole flow controllers and capillary flow controllers. These fixed, fluid-resistance flow controllers usually have a simple structure, no complex parts, low manufacturing and maintenance costs, no moving parts, and a high reliability for long-term use. However, the fluid resistance of the fixed, fluid-resistance flow controller is not adjustable, which makes it difficult to maintain a stable oil film thickness when the load changes. Therefore, it cannot adapt to the dynamically changing needs of the load.

The membrane-type flow controller regulates the fluid resistance by the deformation of a metal film in accordance with load changes, maintaining a constant thickness of the oil film, thus achieving a high stiffness. It is a kind of fluid-resistance variable flow controller. Therefore, in recent years, some new types of membrane restrictors have been mentioned by scholars, of which the following are typical. An island-type membrane restrictor was designed by Zhu et al. [14], which overcame the defect of the inaccurate calculation of membrane deformation and the problem of the membrane being prone to warping during the working process. To improve the stiffness and response characteristics of the bearing, Sawano et al. designed an internal variable restrictor with a thin metal plate [15]. The numerical analysis results showed that internal variable restriction with a thin metal plate could effectively improve the stiffness and response characteristics of the bearing. Furthermore, Lin et al. [16] innovated in the form of throttling and designed a dual-membrane restrictor to improve the stiffness performance of the compensated hydrostatic bearing. In order to address the effects of oil temperature and viscosity changes on throttle performance, Renn et al. [17] designed a spool-type pressure feedback restrictor, which utilized a structural design that combined restrictor holes and adjusting screws. Compared with the traditional membrane-type restrictor, it had the advantage of not being affected by oil viscosity and temperature variation. In terms of design methods for membrane restrictor parameters, Kang et al. [18] proposed a parametric design method for the double-action variable compensation of a membrane-type restrictor, revealing the effect of design parameters on bearing stiffness. Lai et al. [19] investigated the effect of the design parameters of the membrane restrictor on the performance of a hydrostatic opposed tile bearing, given the design process of the high-stiffness membrane restrictor. Hidetoshi et al. investigated the effect of feed hole inlet shape on bearing characteristics [20]. Various types of fluid-resistance variable flow controllers have been mentioned and analyzed for their performance by scholars [21,22,23]. However, the structure of membrane-type flow controllers is much more complex than fixed, fluid-resistance flow controllers (such as capillary tubes and small holes). Therefore, the influence of design parameters, such as the structural dimensions of membrane-type flow controllers, on bearing performance is not clear, and design guidelines for the key dimensional parameters of membrane-type flow controllers need to be further investigated.

**Figure 1:**
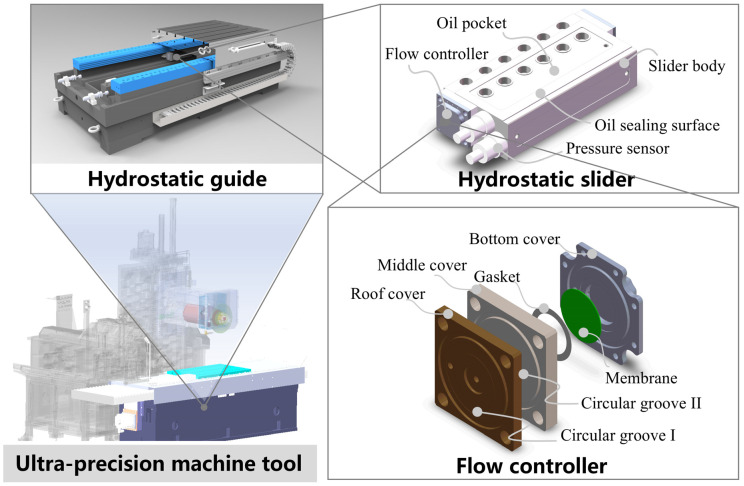
Hydrostatic slider and membrane-type flow controller.

In this study, a membrane-type flow controller with variable fluid resistance is designed (Figure 1), and a theoretical model of the flow controller’s bearing characteristics is established to find the relationship between the structural parameters of the membrane-type flow controller and the bearing performance of the guideway. The accuracy of the theoretical model is verified by fluid–solid coupling simulation and flow rate experiments. According to the theoretical model, the influence of the key structural errors of the membrane-type flow controller on the performance of the hydrostatic guideway is analyzed, design guidelines for membrane-type flow controllers are determined, the key structure of the flow controller is clarified, and the design range of the key structural dimensions is given. The results show that the gasket thickness of the membrane-type flow controller has the greatest influence on the performance of the hydrostatic guide, and it should be ensured that its machining error is less than 0.005 mm. This study serves as a guide for both the design and manufacture of flow controllers and their engineering applications.

## 2. Materials and Methods

### 2.1. Structural Design and Theoretical Modeling of the Flow Controller

**Figure 2:**
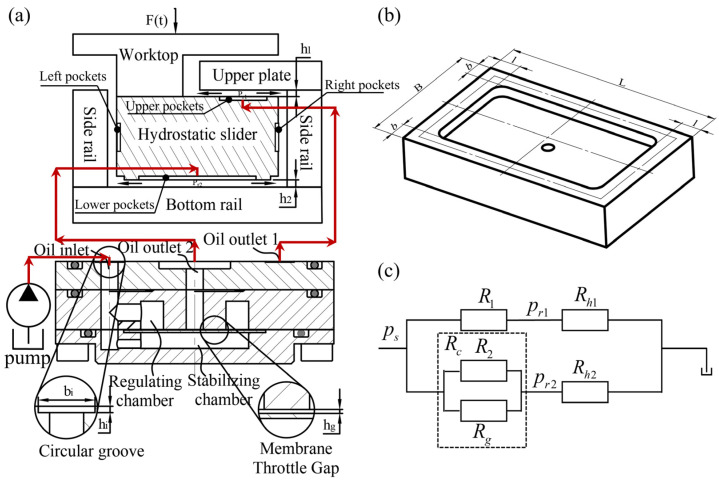
Flow controller principle and fluid resistance diagrams. (**a**) Flow controller working principle, (**b**) the slider oil pocket and sealing oil surface, and (**c**) the fluid resistance diagram.

Hydrostatic guideways can be divided into closed hydrostatic guideways and open hydrostatic guideways according to their different structural forms [6]. This study carries out the structural design of a membrane-type flow controller based on the closed hydrostatic guideway. The closed hydrostatic guideway contains a worktop, hydrostatic slider, upper plate, bottom rail, and side rail, as shown in Figure 2a, in which the hydrostatic slider consists of the flow controller, the pressure sensor, and the slider body, and the lower oil pocket and oil sealing surface are on the slider body. The membrane-type flow controller contains a roof cover, middle cover, bottom cover, membrane, and gasket (Figure 1).

The bottom cover and the membrane form a pressure stabilizing chamber, the middle cover and the membrane form a regulating chamber, and the throttle gap between the membrane and the middle cover boss is controlled by a gasket. Figure 2a shows the working principle of the membrane-type flow controller. The membrane-type flow controller working principle is the membrane in the pressure difference between the stabilizing chamber and the regulating chamber under the action of deformation, and when the external load changes, the pressure difference between the stabilizing chamber and the regulating chamber changes, resulting in a change in the deformation of the membrane. Membrane deformation changes lead to changes in the throttle gap, thereby changing the flow resistance of the fluid and further changes in the flow rate into the slider pocket. Hydraulic oil flows into the oil pocket of the slider under the action of the oil pocket pressure, through the gap between the slider and the surface of the guide out. Usually, the hydrostatic slider and the bottom rail surface form a gap between the surface that is called the oil sealing surface. The slider oil pocket and sealing oil surface are shown in Figure 2b.

When the hydraulic pump supplies oil, the hydrostatic slider floats between the bottom rail and the upper plate due to the hydrostatic pressure of the liquid. An oil film exists between the hydrostatic slider and the bottom rail, and between the hydrostatic slider and the upper plate. When the slider is in force equilibrium, its position remains stable, and the movement of the slider center is constrained by the pressure distribution between the upper/lower and left/right oil pockets. When the external load F(t) increases, the hydrostatic slider force balance is damaged, so the hydrostatic slider undergoes downward movement. The downward movement of the hydrostatic slider leads to a change in the gap between the hydrostatic slider and the bottom rail surface, which leads to a change in the oil resistance of the upper and lower oil pocket. The change in fluid resistance in the upper and lower oil pocket further leads to a change in the flow controller throttle resistance, which means a change in the gap between the restrictor boss and the membrane. The pressure and flow rate of the upper and lower oil pocket of the hydrostatic slider change, causing the hydrostatic slider to move upward to return to its initial position.

According to the above working principle, the fluid resistance diagram of the hydrostatic guideway system is shown in Figure 2c. In this paper, a flow-compensating groove is added to the conventional membrane flow controller, which corresponds to the flow resistance R2 shown in the fluid resistance diagram in Figure 2c. The flow-compensating groove is used to minimize the influence of flow controller manufacturing errors on the bearing performance of the hydrostatic guideway. R2 and Rg are connected in parallel to form a variable fluid resistance that flows to the lower pocket of the slider, and R1 is a fixed fluid resistance that flows to the upper pocket of the slider. Rh1 is the fluid resistance to hydraulic fluid flow from the gap (oil sealing surface) between the slider and the upper plate, and Rh2 is the fluid resistance to hydraulic fluid flow from the gap (oil sealing surface) between the hydrostatic slider and the bottom rail. Based on this fluid resistance diagram, a theoretical model is developed to study the effects of key geometric parameters of the flow controller on the carrying performance of the guideway. The key geometric parameters of interest in this study are film thickness t, gasket thickness h_g_, groove depth h_i_, and the opposite oil pad gap h_0_, which is equal to h_1_ + h_2_ (Figure 2a).

The working process of the hydrostatic guideway is a complex problem. In order to simplify the analysis of the flow characteristics of the hydrostatic guideway, several assumptions should be made according to reference [24,25], as follows:

(1) Assume that the hydrostatic guideway oil pocket pressure is uniformly distributed;
(2) Assume that the guideway and flow controller are rigid;
(3) Assume that the viscosity of the hydraulic oil remains constant and does not change due to temperature, flow rate, and other factors;
(4) Assume that the flow state of the hydraulic oil is laminar;
(5) Assume that the hydraulic fluid is incompressible.

Based on the premise of the above assumptions, the design calculation and practical application of the membrane flow controller, to a certain extent, are approximate and can meet the actual needs of the project.

R_hi_ refers to the fluid resistance of the hydraulic fluid flowing out of the hydrostatic slider oil pocket through the gap between the slider and the rail surface. It can be expressed as follows:

(1) $$R_{hi}=\frac{6l_{i}b_{i}\mu_{t}}{{h_{i}}^{3}l_{i}(L_{i}-l_{i})+b_{i}(B_{i}-b_{i})]}=\frac{R_{hi\_k}}{{h_{i}}^{3}}$$

where i=1,2;

$\mu_{t}$—Dynamic viscosity of hydraulic oil;
$h_{i}$—Thickness of oil film in upper and lower oil pocket;
L,l,B,b—Slide oil seal surface size parameters;
$R_{hi\_k}$—Oil pocket liquid resistance coefficient.

The fluid resistance of the membrane can be expressed as follows:

(2) $$R_{g}=\frac{6\mu_{t}\ln\left(\frac{r_{g2}}{r_{g1}}\right)}{\pi {\left(h_{g}\right)}^{3}}=\frac{R_{g\_k}}{{h_{g}}^{3}}$$

In the above equation, h_g_ refers to the resistance gap between the boss and the membrane, r_g1_ and r_g2_ refer to the inner and outer diameters of the boss, and R_g_k_ refers to the liquid resistance coefficient of the membrane.

Based on Figure 2c, it can be seen that R_c_ is the parallel liquid resistance of R_2_ and R_g_, so R_c_ can be expressed as follows:

(3) $$R_{c}=\frac{R_{2}R_{g}}{R_{2}+R_{g}}$$

Meanwhile, the liquid resistance ratio λ_1_ is defined as the ratio of liquid resistance R_1_ to liquid resistance R_h1_, λ_210_ as the ratio of liquid resistance R_2_ to liquid resistance R_h2_, λ_220_ as the ratio of liquid resistance R_c_ to liquid resistance R_h2_, and λ_2_ as the ratio of liquid resistance R_c_ to R_h2_. β refers to the throttling ratio, which is the ratio of the oil supply pressure P_s_ to the oil chamber pressure P_r_. So, the upper and lower pocket pressures of the slider can be expressed as Equations (8) and (11).

(4) $$\lambda_{210}=\frac{R_{2}}{R_{h2}}=\frac{R_{2}{\left(h_{2}\right)}^{3}}{R_{h2\_k}}$$

(5) $$\lambda_{220}=\frac{R_{g}}{R_{h2}}=\frac{R_{g\_k}{\left(h_{2}\right)}^{3}}{{\left(h_{g}\right)}^{3}R_{h2\_k}}$$

(6) $$\lambda_{2}=\frac{R_{c}}{R_{h2}}=\frac{\frac{R_{2}R_{g}}{R_{2}+R_{g}}}{R_{h2}}=\frac{R_{2}R_{g\_k}{\left(h_{2}\right)}^{3}}{R_{2}{\left(h_{g}\right)}^{3}R_{h2\_k}+R_{g\_k}R_{h2\_k}}$$

(7) $$\beta_{2}=\lambda_{2}+1=\frac{R_{2}R_{g\_k}{\left(h_{2}\right)}^{3}}{R_{2}{\left(h_{g}\right)}^{3}R_{h2\_k}+R_{g\_k}R_{h2\_k}}+1$$

(8) $$P_{r2}=\frac{P_{s}R_{h2}}{R_{c}+R_{h2}}=\frac{P_{s}}{\frac{R_{2}R_{g\_k}{\left(h_{2}\right)}^{3}}{R_{2}{\left(h_{g}\right)}^{3}R_{h2\_k}+R_{g\_k}R_{h2\_k}}+1}=\frac{P_{s}}{\beta_{2}}$$

(9) $$\lambda_{1}=\frac{R_{1}}{R_{h1}}=\frac{R_{1}{\left(h_{1}\right)}^{3}}{R_{h1\_k}}$$

(10) $$\beta_{1}=\lambda_{1}+1=\frac{R_{1}{\left(h_{1}\right)}^{3}}{R_{h1\_k}}+1$$

(11) $$P_{r1}=\frac{P_{s}R_{h1}}{R_{1}+R_{h1}}=\frac{P_{s}}{\frac{R_{1}}{R_{h1}}+\frac{R_{h1}}{R_{h1}}}=\frac{P_{s}}{\lambda_{1}+1}=\frac{P_{s}}{\beta_{1}}$$

Based on the above equation, the initial flow rate Q_10_ of the upper oil pocket on the guideway slide can be expressed as follows:

(12) $$Q_{10}=\frac{p_{s}}{R_{1}+R_{h10}\;}=\frac{p_{s}-p_{r10}}{R_{1}}=\frac{p_{r10}}{R_{h10}}=\frac{p_{r10}h_{10}^{3}}{R_{h1\_k}}$$

The initial flow rate Q_20_ in the lower oil pocket of the guideway slide is as follows:

(13) $$Q_{20}=\frac{p_{s}}{R_{0}}=\frac{p_{s}-p_{r20}}{R_{c0}}=\frac{p_{r20}}{R_{h20}}=\frac{p_{r20}h_{20}^{3}}{R_{h2\_k}}$$

where $R_{0}=R_{c0}+R_{h20}$, $R_{c0}=\frac{R_{2}R_{g0}}{R_{2}+R_{g0}}$. In the working condition, the flow rate of the oil pocket on the guideway slide is as follows:

(14) $$Q_{1}=\frac{p_{s}}{R_{1}+R_{h1}\;}=\frac{p_{s}-p_{r1}}{R_{1}}=\frac{p_{r1}}{R_{h1}}=\frac{p_{r1}{\left(h_{10}+e\right)}^{3}}{R_{h1\_k}}$$

The initial flow rate in the lower oil pocket of the guideway slide is as follows:

(15) $$Q_{2}=\frac{p_{s}}{R}=\frac{p_{s}-p_{r2}}{R_{c}}=\frac{p_{r2}}{R_{h2}}=\frac{p_{r2}{\left(h_{20}-e\right)}^{3}}{R_{h2\_k}}$$

**Figure 3:**
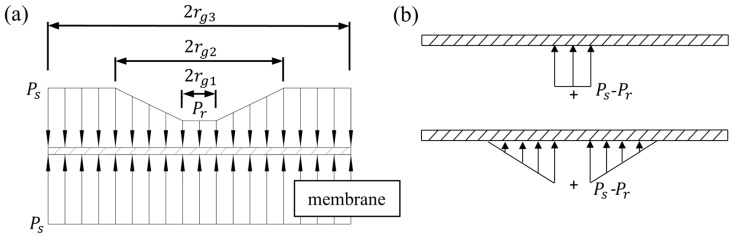
Pressure distribution over the membrane of the flow controller. (**a**) Overall, (**b**) Decomposed.

As shown in Figure 3, due to the throttling effect of the membrane gap, the pressure distribution on the membrane of the flow controller is not uniform. In this regard, scholars have conducted a lot of work to calculate the membrane’s deformation accurately. In this paper, assuming that the membrane boundary is fixed, we adopt the superposition method to determine the deformation of the membrane. The specific derivation process is as follows:

(16) $$\begin{array}{cc} \delta \left(F\right) & =\delta_{A}+\delta_{B}+\delta_{C}\\ \delta_{A} & =\frac{12{\left({r_{g3}}^{2}-{r_{g1}}^{2}\right)}^{2}\left(1-m^{2}\right)\left(P_{s}-P_{t}\right)}{64Et^{3}}=K_{1}\left(p_{s}-p_{t}\right)\\ \delta_{B} & =\int_{0}^{r_{g1}}\frac{\left(P_{t}-P_{r}\right)r}{8D}\left[\begin{array}{l} 2\left(r^{2}+{r_{g1}}^{2}\right)\ln\left(\frac{r_{g1}}{r_{g3}}\right)+\\ \frac{\left(r^{2}+{r_{g3}}^{2}\right)\left({r_{g3}}^{2}-{r_{g1}}^{2}\right)}{{r_{g3}}^{2}} \end{array}\right]dr\\ & =K_{2}\left(p_{t}-p_{r}\right)\\ \delta_{C} & =\int_{r_{g1}}^{r_{g2}}\begin{array}{l} \frac{\left(\left(P_{t}-P_{r}\right)-\frac{\left(P_{t}-P_{r}\right)\left(r-r_{g1}\right)}{r_{g2}-r_{g1}}\right)r\cdot }{8D}\\ \left[2\left(r^{2}+{r_{g1}}^{2}\right)\ln\left(\frac{r}{r_{g3}}\right)+\frac{\left({r_{g3}}^{2}+{r_{g1}}^{2}\right)\left({r_{g3}}^{2}-r^{2}\right)}{{r_{g3}}^{2}}\right] \end{array}dr\\ & =K_{3}\left(p_{t}-p_{r}\right)\\ D & =\frac{Et^{3}}{12\left(1-m^{2}\right)}\\ \delta \left(F\right) & =\left[\left(K_{2}+K_{3}\right)\lambda_{2}+K_{1}\lambda_{1}\right]p_{r} \end{array}$$

The force balance equation for the working state of the guideway slide is as follows:

(17) $$\frac{P_{s}A_{2}}{\beta_{2}}-\frac{P_{s}A_{1}}{\beta_{1}}-mg=0$$

The membrane deformation equation is as follows:

(18) $$\left(K_{1}+K_{3}\right)p_{r2}\lambda_{2}-h_{g0}+h_{g}=0$$

Based on Equations (17) and (18), theoretical modeling of the bearing characteristics of a single-slide closed-type guideway is prepared in matlab. The effect of the flow controller’s design parameters on the guideway performance is investigated by varying the guide-rail-opposed oil pad gap, membrane thickness, shim thickness, and groove depth.

### 2.2. Simulation Modeling of Bidirectional Fluid-Solid Coupling

In order to further verify the accuracy of the theoretical model, a fluid–solid coupling simulation model of the flow controller is established by ANSYS 2022R1. The fluid–solid coupling simulation is classified into unidirectional fluid–solid coupling simulation and bidirectional fluid–solid coupling simulation [26]. Because the deformation of the membrane in the flow controller has a great influence on the fluid, bidirectional fluid–solid coupling simulation is used in this paper. That is, at the same time, we consider the effect of fluid motion on solid deformation and the effect of solid deformation on fluid motion.

In the bidirectional fluid-solid coupling simulation, there are interactions between the fluid and the solid, which require a large number of calculations, so the ortho-hexahedral structural meshing technique is adopted. In the structured meshing process, in order to improve the computational efficiency and accuracy, the adaptive meshing technique is adopted, which focuses on ensuring that the number of mesh layers at the gap of the film is greater than 15 (the mesh size is less than 6 μm). The adaptive meshing technique is based on the characteristics and needs of the simulation area, using smaller grid cells where a higher resolution is needed and larger grid cells in areas where a high resolution is not required.

**Figure 4:**
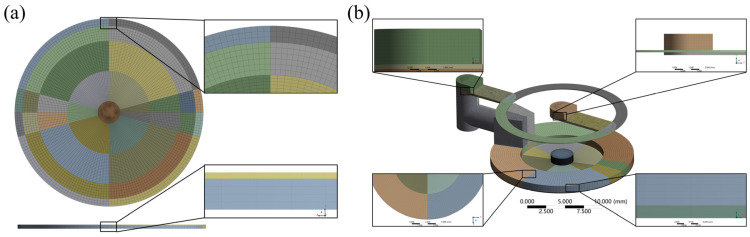
Ortho-hexahedral structured meshing results. (**a**) Solid domain. (**b**) Fluid domain.

The results of the ortho-hexahedral structured meshing of the solid and fluid domains of the flow controller are shown in Figure 4. The number of grid cells in the fluid domain is 523,060 in total, of which the average cell mass is 0.49, the average orthogonal mass is 0.88, and the average skewness is 0.14; the number of grid cells in the solid domain is 44,600 in total, of which the average cell mass is 0.69, the average orthogonal mass is 0.99, and the average skewness is 0.03.

**Figure 5:**
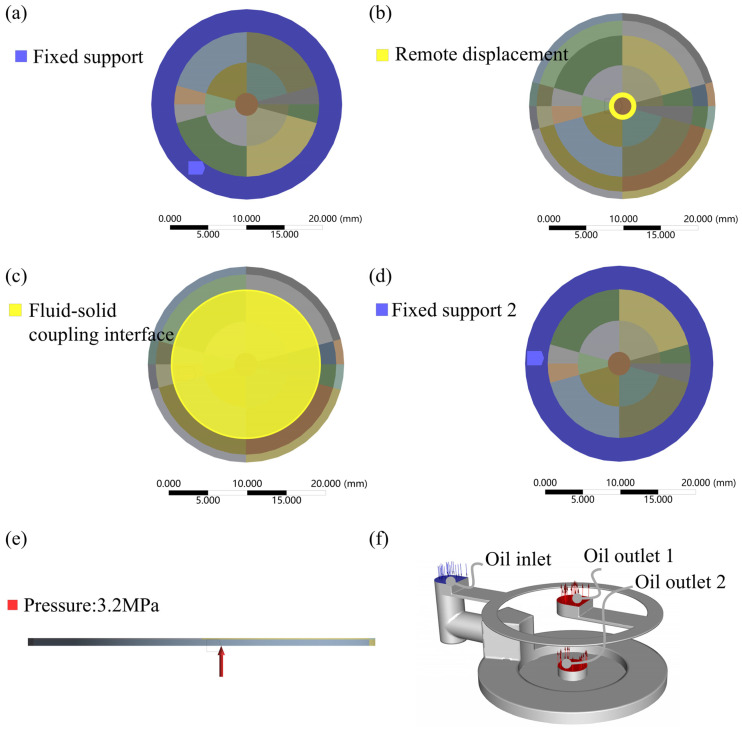
Simulation setup. (**a**) Fixed support, (**b**) remote displacement, (**c**) fluid–solid coupling interface, (**d**) fixed support2, (**e**) pressure, and (**f**) fluid domain inlet and outlet ports.

The mechanical module in ANSYS workbench is set up for solving the solid domain, and the material of the membrane in the solid domain is 65 Mn spring steel, with the density set to 7.85 × 10^−6^ kg/mm^3^, Young’s modulus set to 2 × 10^5^ MPa, Poisson’s ratio set to 0.3, and shear modulus set to 76,923 MPa. As shown in Figure 5a–d, the outer annular band portions of the upper and lower surfaces of the membrane are provided with fixed support constraints; as shown in Figure 5e, the stabilizing chamber pressure of 3.2 MPa is simplified to be applied to the lower surface of the membrane as a constant pressure; the center of the membrane is set to be a remotely displaced constraint, as shown in Figure 5b; and the fluid–solid coupling interface is set to be the region in contact with the fluid, as shown in Figure 5c. The Fluent module in ANSYS workbench is set up to solve the fluid domain. The laminar flow model is selected for the fluid simulation model, and the fluid material is set as No.32 lubricating oil with a density of 960 kg/m^3^ and a viscosity of 0.1214 kg/(m.s). The inlet and outlet ports are set as shown in Figure 5f. To reduce the amount of modeling calculations, two ports are set, where outlet 2 corresponds to membrane gap variable throttling and outlet 1 corresponds to flow-compensated groove fixed throttling. The dynamic mesh method is set to smoothing, and the fluid–solid coupling surface is the bottom surface, which is the contact surface between the fluid and the membrane. The solution method is set to SIMPLEC, monitoring the pressure and flow rate at the inlet and outlet ports, with the residual set to 0.0001, convergence conditions set to a flow stopping criterion of 0.0001, a pressure stopping criterion of 0.001, and the calculation set to an iteration number of 500. The pressure and flow rate of the inlet and outlet ports are monitored and initialized by the hybrid initialization method.

After the static structure and fluid flow module is set up, the data are transferred to the system coupling module setup, and the flow–solid coupling solution is set up in the system coupling module.

### 2.3. Flow and Bearing Characteristic Test Platforms

**Figure 6:**
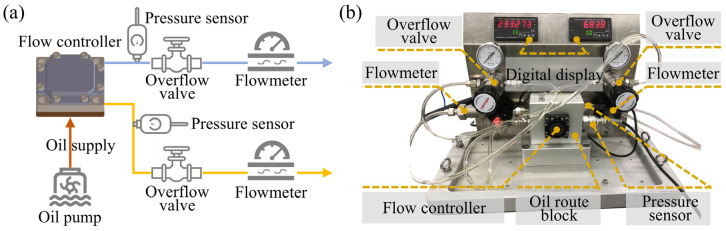
Test platform for flow rate of flow controller. (**a**) Experimental principle and (**b**) actual platform.

**Figure 7:**
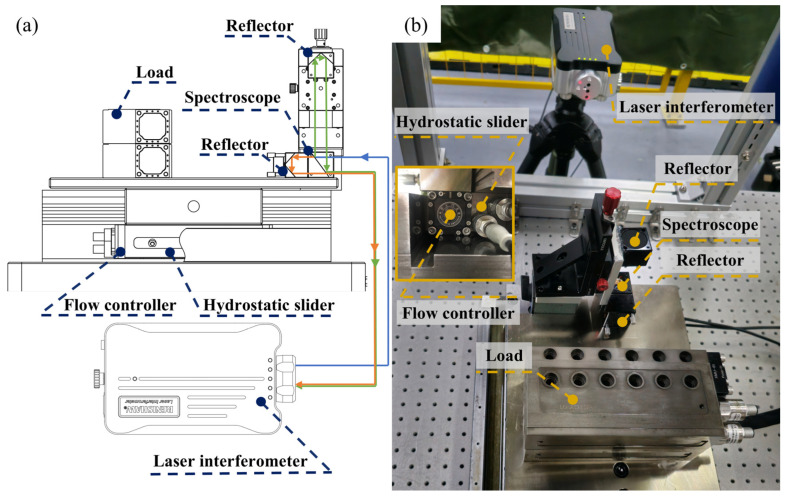
Test platform for bearing characteristics of flow controller. (**a**) Experimental principle and (**b**) actual platform.

In order to verify the accuracy of the theoretical model and simulation model of the membrane-type flow controller, we build a flow rate test platform (Figure 6) and a bearing characteristic test platform (Figure 7) for the flow controller. These are used to carry out flow experiments and bearing characteristic experiments of the flow controller.

The flow rate experimental principle of the flow controller is shown in Figure 6a, and the actual platform is shown in Figure 6b. The flow rate test platform consists of a flow controller, oil route block, overflow valve, flowmeter, digital display, pressure sensor, etc. Hydraulic oil with a constant pressure output from the oil pump flows into the oil route block through the inlet of the oil route block. Hydraulic oil flows into the flow controller inlet through the internal flow path of the oil route block and is output from the hydraulic connector after being throttled by the flow controller. The output hydraulic oil flows through the flowmeter, and the flow rate of hydraulic oil through the flowmeter can be displayed through the digital display meter. By changing the oil pump supply pressure, the initial flow rate of the flow controller can be tested at different supply pressures. By replacing the flow controller on the oil route block, it is possible to test the initial flow of different flow controllers at the same supply pressure.

The bearing characteristics test platform of the flow controller is shown in Figure 7, which consists of a Renishaw laser interferometer, closed hydrostatic guideway, hydrostatic slider, flow controller, and load. The reflector of the interferometer is installed on the upper plate and the spectroscope of the interferometer is mounted on the table. Loading is carried out on the table under the condition of a stable oil supply to the guideway, and the laser interferometer is used to measures the change in film thickness in the oil pocket under the hydrostatic slide before and after loading.

## 3. Results and Discussion

### 3.1. The Influence of Design Parameters on Bearing Characteristics

The design parameters of interest in this study are membrane thickness t, gasket thickness h_g_, groove depth h_i_, and the opposite oil pad gap h_0_. Therefore, the following discussion addresses the above four design parameters.

**Figure 8:**
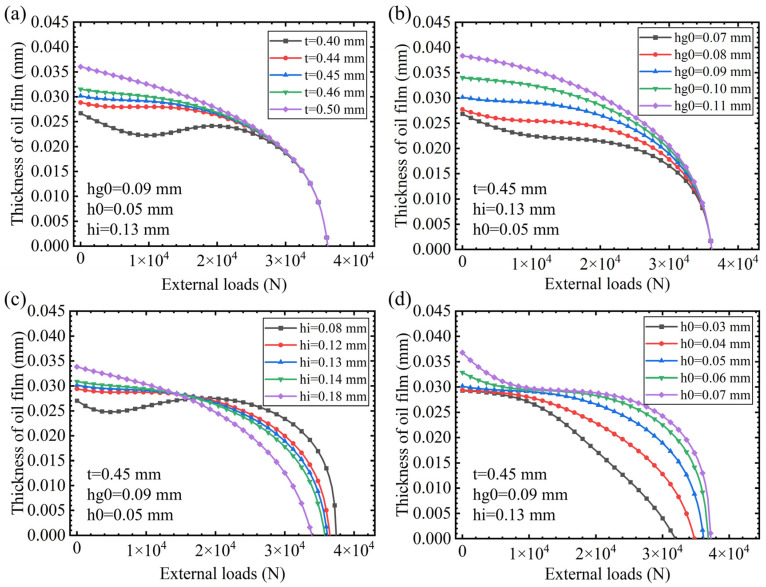
Influence of flow controller design parameters on bearing characteristics. (**a**) Membrane thickness, (**b**) gasket thickness, (**c**) groove depth, and (**d**) opposite oil pad gap.

The elastic membrane is the key part of the membrane flow controller, which can generate elastic deformation to change the fluid resistance of the flow controller when the external load is changed, thus giving the hydrostatic guideway a greater stiffness. The thickness and diameter of the elastic membrane are closely related to its deformation, and due to the restriction of the size of the flow controller, the diameter of the membrane is fixed, so the thickness of the membrane is an important parameter that can be adjusted when designing the restrictor. Figure 8a indicates that under zero external load, with an increase in the elastic membrane thickness, the thickness of the oil film in the lower oil pocket also increases. This is because the greater the thickness of the elastic membrane, the smaller its deformation under the pressure of the stabilizing chamber, the greater the throttle gap between the elastic membrane and the boss, the smaller the throttle fluid resistance Rg, and the greater the flow rate of the lower oil chamber. Further analysis of the impact of film thickness on the bearing performance of the hydrostatic guide can be seen. When the deviation between the actual membrane thickness and the design thickness of the membrane is less than 0.01 mm, the impact on the hydrostatic support is not significant, and it only affects the relative position of the balance of the hydrostatic support, which leads to inconsistency in the membrane thickness of the upper and lower oil pockets. When the elastic membrane manufacturing error is greater than 0.05 mm, the impact of the membrane thickness of the hydrostatic guide on bearing characteristics is more obvious, not only affecting the relative position of the hydrostatic guide balance, but also directly affecting the oil film stiffness. For example, when the thickness of the elastic membrane is less than 0.40 mm, with an increase in load, the thickness of the oil film in the lower oil pocket is oscillating and unstable, incurring a negative stiffness. The reason for this is that as the film thickness decreases, the initial deformation of the film increases, resulting in a very small throttling gap between the film and the throttling boss, with almost no hydraulic oil flowing through the throttling gap between the film and the boss. When the external load increases, the film throttling gap widens, allowing more hydraulic oil to flow through the film throttling gap into the oil chamber, thereby increasing the thickness of the guide rail oil film. When the load continues to increase, the film no longer deforms, effectively functioning as a fixed restrictor, causing the oil film thickness to drop sharply. Therefore, in engineering applications, it should be ensured that the membrane thickness is taken to be 0.45 ± 0.01 mm. In addition, for closed multi-hydrostatic support guideway systems, it should be ensured that the membrane thickness manufacturing error is consistent in the same system, so as to avoid hydrostatic sliding part deflection due to deviations in the balance position of the support, which will result in hydrostatic guideway system failures.

The throttle gap of the flow controller refers to the initial throttle gap between the membrane and the boss. The initial throttle gap between the membrane and the boss is controlled by the gasket in the membrane flow controller, which directly affects the initial throttle flow resistance. The thickness of the gasket should be an appropriate size. If the thickness is too large, the membrane deformation for the throttle gap is no longer significant, and the membrane flow controller is equivalent to a fixed, flow-resistance flow controller. If the thickness is too small, then the oil in the stabilizing chamber under the action of membrane deformation greater than the throttle gap cannot be in normal circulation. Figure 8b demonstrates that, under zero external load, the thickness of the oil film in the lower oil pocket increases as the thickness of the gasket (throttle gap) increases. This is due to the fact that the greater the shim thickness, the greater the throttle gap between the elastic film and the boss, the smaller the throttle fluid resistance, and the greater the lower oil pocket flow. Further analysis shows that the gasket thickness has a significant effect on the bearing performance of the hydrostatic guide. When the deviation between the actual thickness of the gasket and the design value of the gasket thickness is greater than 0.01 mm, the oil film thickness changes significantly under the external load, indicating that the hydrostatic guide has a poor stiffness. Therefore, in engineering applications, it should be ensured that the gasket thickness is taken as 0.09 ± 0.005 mm.

The depth of the groove of the flow controller is the depth of the circular groove h_I_ corresponding to the upper oil pocket of the slider and the depth of the circular groove h_II_ corresponding to the lower oil pocket of the slider. h_I_ affects the throttle fluid resistance R_1_ in the upper oil pocket of the flow controller, and h_II_ affects the throttle fluid resistance R_2_ in the lower oil pocket of the flow controller. The depth of the groove of the flow controller is very important in controlling the lower oil pocket flow and the upper oil pocket flow of the flow controller. Figure 8c indicates that, under zero external load, the oil film thickness in the lower oil pocket increases as the depth of the groove increases. This is due to the fact that the larger the groove depth, the smaller the groove throttling fluid resistance, and, therefore, the flow rate of the lower oil pocket is larger. When the manufacturing error of the groove depth is less than or equal to ±0.01 mm, the impact of the manufacturing error of the groove depth on the hydrostatic support can be ignored. When the manufacturing error of the groove depth is greater than or equal to ±0.05 mm, the effect of the manufacturing error of the groove depth on the hydrostatic support is significant. In addition, when the manufacturing error is positive, the oil film thickness of the lower oil pocket shows a significant negative correlation with the external load. When the groove depth manufacturing error is negative, the oil film thickness of the lower oil pocket first fluctuates with an increase in load and then shows a negative correlation trend. This is because when the manufacturing error of the groove depth is negative, the groove throttling fluid resistance becomes larger, and the compensation effect of the groove flow on the membrane throttling gap is weakened. Due to the characteristics of the membrane-type flow controller, a negative stiffness occurs. Therefore, in engineering applications, it should be ensured that the groove depth is 0.13 ± 0.01 mm.

The opposite oil pad gap refers to the closed guide on the upper pocket oil film thickness and the lower oil pocket oil film thickness of the sum, and it depends on the machining accuracy and assembly accuracy of the guide. As can be seen from Figure 8d, the deviation of the opposed oil pad gap significantly affects the stiffness of the hydrostatic guideway and the initial equilibrium position of the slide. Under zero external load conditions, the larger the opposite oil pad gap, the smaller the flow resistance at the sealing surface and the greater the flow rate, therefore leading to a greater oil film thickness. The larger the deviation of the actual value of the opposed oil pad gap from the design value, the worse the initial stiffness of the hydrostatic guideway, so in actual processing, focus should be on controlling the oil pad gap of the hydrostatic guideway, and the dimensional tolerance should not be too large. At the same time, it should be ensured that the gap manufacturing error of the opposite oil pad is in the negative deviation. In this case, although the overall stiffness of the hydrostatic guideway system has decreased, the initial stiffness of the oil film is still able to maintain a good state. Therefore, in engineering applications, it should be ensured that the opposite oil pad gap is taken as $0.05_{-0.02}^{0}$ mm.

In summary, in all four subfigures of Figure 8, the oil film thickness decreases as the external load increases, while different parameters lead to varying rates of change in the oil film thickness. Under the same load, changes in parameters t, hg0, and h0 have a relatively consistent effect on the oil film thickness, and increasing these parameters results in an increase in the oil film thickness. In contrast, the influence of parameter hi is more complex and load-dependent. Additionally, a comparison of Figure 8a–d reveals that machining errors in the gasket thickness have the most significant impact on the bearing characteristic curves of the oil film. Noticeable changes in the bearing characteristic curves occur when the gasket thickness error is within 0.09 ± 0.01 mm. For the film thickness, groove depth, and opposed oil pad gap, significant changes in the bearing characteristic curves are observed when the errors are within 0.45 ± 0.05 mm, 0.13 ± 0.05 mm, and 0.05 + 0.01 mm. Through the above analysis, we have optimized and selected the design parameters for the flow controller, as presented in Table 1.

### 3.2. Initial Flow Rate of the Flow Controller

**Figure 9:**
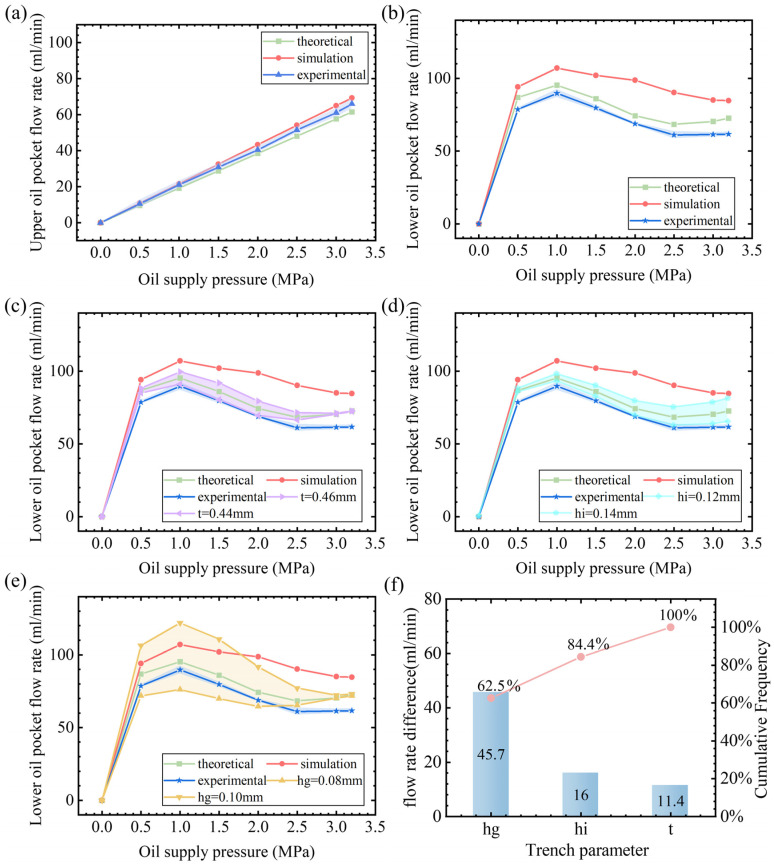
Initial flow rate of the membrane restrictor. (**a**) Upper oil pocket, (**b**) lower oil pocket, (**c**) the effect of membrane thickness manufacturing errors on flow rate, (**d**) the effect of groove depth manufacturing errors on flow, (**e**) the effect of gasket thickness manufacturing errors on flow, and (**f**) Pareto chart of parameters.

Figure 9a presents a comparison of the theoretical, simulation, and experimental values of the flow controller’s fixed fluid resistance oil path (upper oil pocket) flow rate at different oil supply pressures. It can be clearly seen that the theoretical, simulation, and experimental values of the flow rate of the fixed fluid resistance oil circuit show a consistent linear growth trend, indicating that the flow rate of the fixed fluid resistance oil circuit shows a linear relationship with an increase in pressure within the range of 0–3.2 MPa oil supply pressure, and the deviation between the theoretical, simulation, and experimental values is small, which verifies the validity of the theoretical model and the fluid–solid coupling model. Figure 9b demonstrates the variation in the initial flow rate in the lower pocket of the membrane flow controller with the oil supply pressure. Obviously, the flow rate of the lower pocket of the membrane type flow controller has a nonlinear relationship with the oil supply pressure. Under the conditions of membrane thickness t = 0.45 mm, gasket thickness hg = 0.09 mm, and groove depth hi = 0.13 mm, the three curves obtained from theoretical calculations, fluid–solid coupling simulations, and experimental tests show the same trend, which increases and then decreases to a certain value and remains stable. The deviations in the values of the three curves are due to manufacturing errors in film thickness, shim thickness, and groove depth, as well as assumptions made during simulation. From Figure 9c, it can be seen that the light-purple region is the range of the lower oil pocket flow rate calculated by the theoretical model considering a film thickness manufacturing error of t = 0.45 ± 0.01 mm. In Figure 9d, it can be seen that the light-cyan area is the lower oil pocket flow range calculated by the theoretical model considering a groove depth machining error of hi = 0.13 ± 0.01 mm. Figure 9e shows that the light-yellow area is the lower oil pocket flow range calculated by the theoretical model considering a gasket thickness manufacturing error of hg = 0.09 ± 0.01 mm. In summary, the trends of the theoretical calculations, fluid–solid coupling simulations, and experimental values of the flow rate are consistent, and the numerical deviations are all within the range of the flow rate deviation caused by the machining error of the flow controller. Therefore, the theoretical model established in this study is sufficiently accurate. Comparison of Figure 9c–e shows that the range of flow fluctuation due to the gasket thickness machining error is the largest for the same machining error. In addition, the Pareto analysis in Figure 9f shows that the gasket thickness dominates the sensitivity of the initial flow in the lower oil pocket, accounting for 62.5% of the total variation. Groove depth ranks second with 21.9% of the total variation, and the groove depth has a smaller effect on the flow rate with 15.6% of the total variation. Therefore, gasket thickness is the most sensitive parameter to ensure that machining error is less than 0.005 mm.

**Figure 10:**
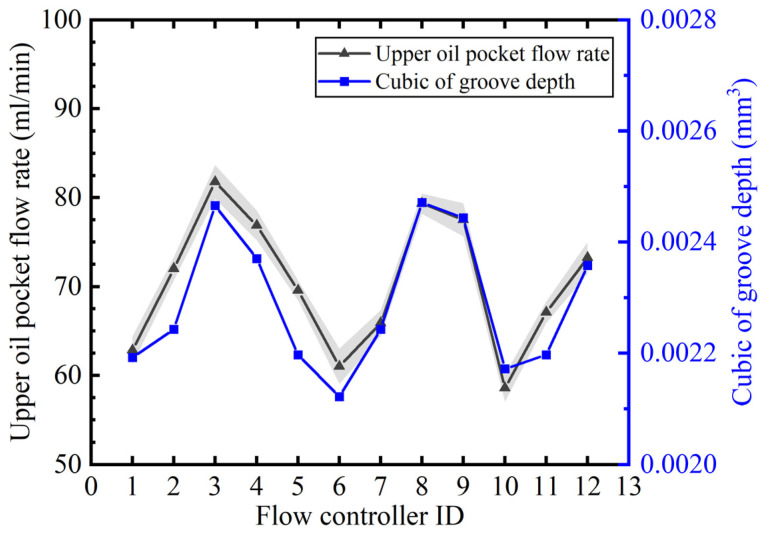
Correspondence between flow and cubic depth of the trench.

In the theoretical model, we see that the fluid resistance of the groove is inversely proportional to the third power of the groove depth, and the flow rate is proportional to the third power of the depth. We manufacture a set of flow controllers, measure the groove depth of the flow controllers by 518-351DC height gauge, and measure the flow rate of the flow controllers at the oil supply pressure of 3.2 Mpa by the flow experimental platform. Figure 10 compares the distribution trends of the flow rates of the 12 pieces of flow controllers with their corresponding groove depth cubic, and the results show that the flow rate and the cubic change trend of the groove depth are consistent and the flow rate is positively correlated with the cubic of the groove depth, which illustrates the accuracy of the theoretical calculation of flow rate of the fixed groove of the throttle.

### 3.3. Bearing Characteristics of Closed Guideway

**Figure 11:**
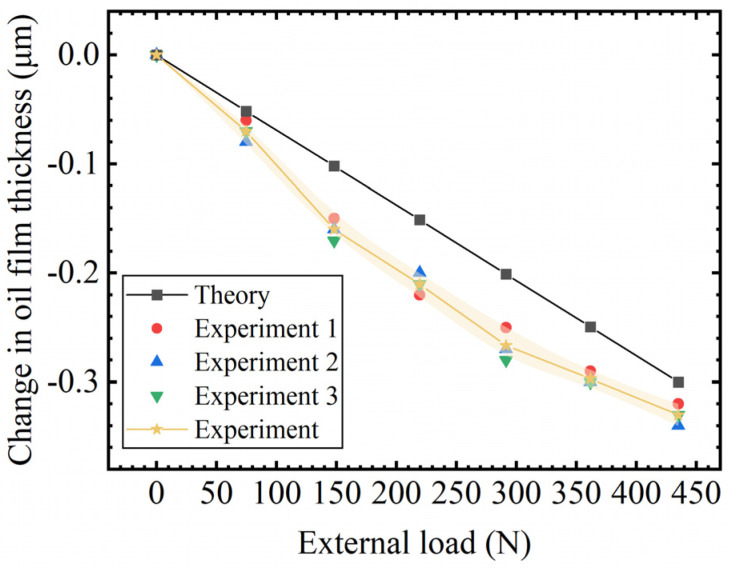
Test results of flow controller bearing characteristics.

Figure 11 shows the load-bearing characteristic curve of the membrane-type flow controller designed in this paper, which is the change in the oil film thickness under the action of external load. From the overall trend, the oil film thickness shows a monotonical decrease with an increase in external load. The theoretical calculation results and experimental data, as a whole, show a high degree of consistency, although there is a certain degree of dispersion in the experimental data, the deviation is not large, and the overall trend is consistent with the results of the theoretical calculation value, which verifies the validity of the theoretical model and the stability of the performance of the flow controller.

## 4. Conclusions

In this study, a membrane-type flow controller with variable fluid resistance is designed, which innovatively provides a new approach for enhancing the bearing performance of hydrostatic guides. The influence of the design parameters of the membrane-type flow controller on the bearing performance of the hydrostatic guide is investigated, the key structure of the flow controller is clarified, and the design range of the key structure dimensions is given.

Based on the fluid resistance principle diagram of the membrane flow controller, a theoretical model of the load-bearing characteristics is established. Through the finite element software, a bidirectional fluid–solid coupling simulation model of the membrane flow controller is established, and an experimental verification platform for flow rate and load-bearing characteristics is constructed. The experimental results of flow rate and load-bearing characteristics verify the accuracy of the theoretical model and the simulation model. This provides a more reliable method for predicting and optimizing the performance of the flow controller.

The analysis results show that the gasket thickness of the membrane-type flow controller has the greatest influence on the performance of the hydrostatic guideway, and it should be ensured that the thickness of the gasket is in the range of 0.09 ± 0.005 mm. In addition, the design reference range for the thickness of the membrane is 0.45 ± 0.01 mm, that for the depth of the grooves is 0.13 ± 0.01 mm, and that for the gap of the opposed oil pad is 0.03–0.05 mm. These precise design ranges can effectively guide the engineering application of the membrane flow controller, improve the bearing capacity and stability of the hydrostatic guide, and have important practical value for promoting the development of related fields.

## Figures and Tables

**Table 1:** Optimized design parameters for membrane-type flow controller.

Throttle Tabs/mm			Membrane Thickness/mm	Membrane Material /MPa		Gasket Thickness /mm
*r_g_*_1_	*r_g_*_2_	*R_g_*_3_	*t*	*E*	*m*	*h_g_*_0_
1.5	5.5	9.5	0.45	2 × 10^5^	0.3	0.09
Circular groove I/mm			Circular groove II/mm			Opposite oil pad gap/mm
*b*_I_	*h*_I_	*r*_I_	*b*_II_	*h*_II_	*r*_II_	*h*_0_
5	0.13	15	1.5	0.13	8	0.05

## Data Availability

The data that support the findings of this study are available from the corresponding author upon reasonable request.
